# Developing a random forest algorithm to identify patent foramen ovale and atrial septal defects in Ontario administrative databases

**DOI:** 10.1186/s12911-022-01837-2

**Published:** 2022-04-06

**Authors:** Laura Oliva, Eric Horlick, Bo Wang, Ella Huszti, Ruth Hall, Lusine Abrahamyan

**Affiliations:** 1 Institute of Health Policy, Management and Evaluation (IHPME), University of Toronto, Toronto, ON Canada; 2 Peter Munk Cardiac Centre, Toronto General Hospital, University Health Network (UHN), Toronto, ON Canada; 3 Department of Medicine, University of Toronto, Toronto, ON Canada; 4 Techna Institute, UHN, Toronto, ON Canada; 5 CIFAR, Toronto, ON Canada; 6 Biostatistics Research Unit (BRU) Toronto General Hospital Research Institute, UHN, Toronto, ON Canada; 7 ICES, Toronto, ON Canada; 8 Toronto General Hospital Research Institute, UHN, 10th Floor Eaton North, Room 237, 200 Elizabeth Street, Toronto, ON M5G 2C4 Canada; 9 Toronto Health Economics and Technology Assessment (THETA) Collaborative, UHN, Toronto, ON Canada

**Keywords:** Foramen ovale, Patent, Machine learning, Septal occluder device, Septal defects, Atrial

## Abstract

**Purpose:**

Routinely collected administrative data is widely used for population-based research. However, although clinically very different, atrial septal defects (ASD) and patent foramen ovale (PFO) share a single diagnostic code (ICD-9: 745.5, ICD-10: Q21.1). Using machine-learning based approaches, we developed and validated an algorithm to differentiate between PFO and ASD patient populations within healthcare administrative data.

**Methods:**

Using data housed at ICES, we identified patients who underwent transcatheter closure in Ontario between October 2002 and December 2017 using a Canadian Classification of Interventions code (1HN80GPFL, N = 4680). A novel random forest model was developed using demographic and clinical information to differentiate those who underwent transcatheter closure for PFO or ASD. Those patients who had undergone transcatheter closure and had records in the CorHealth Ontario cardiac procedure registry (N = 1482) were used as the reference standard. Several algorithms were tested and evaluated for accuracy, sensitivity, and specificity. Variable importance was examined via mean decrease in Gini index.

**Results:**

We tested 7 models in total. The final model included 24 variables, including demographic, comorbidity, and procedural information. After hyperparameter tuning, the final model achieved 0.76 accuracy, 0.76 sensitivity, and 0.75 specificity. Patient age group had the greatest influence on node impurity, and thus ranked highest in variable importance.

**Conclusions:**

Our random forest classification method achieved reasonable accuracy in identifying PFO and ASD closure in administrative data. The algorithm can now be applied to evaluate long term PFO and ASD closure outcomes in Ontario, pending future external validation studies to further test the algorithm.

**Supplementary Information:**

The online version contains supplementary material available at 10.1186/s12911-022-01837-2.

## Introduction

Affecting up to 25% of adults, patent foramen ovale (PFO) is a condition that results from the post-partum persistence of a passage, the foramen ovale, in the atrial septum [1–3] PFO increases the risk of a number of adverse clinical outcomes including cryptogenic stroke [1, 4]. The current standard of care for selected cryptogenic stroke patients with PFO is a transcatheter closure (TC) procedure, where a double disk occlusion device is implanted into the septum [5, 6]. Due to its minimally invasive nature and support from randomized controlled trials (RCTs) for decreasing the risk of repeat stroke and other adverse cardiovascular outcomes, implantation of transcatheter devices is widely considered to be a safe, effective, and economical option for PFO closure [1, 4, 7, 8]. Given that much of the widespread implementation of transcatheter closure is based upon RCTs, there is a growing interest in the evaluation of long-term post-closure outcomes using population-level observational data [9, 10].

Administrative health data provides a rich source of population-level information [11]. Routinely collected for billing and other administrative purposes, health administrative data contains demographic, procedural, and diagnostic data, typically coded based on International Statistical Classification of Diseases (ICD), and allow for a cost-effective means to study health care delivery, quality, costs, effectiveness and safety [10–14]. However, the use of healthcare administrative databases for research comes with the caveat that there is a lack of granularity in clinical detail [10, 11]. This results in challenges with describing and differentiating some conditions of interest relying solely on diagnostic or procedural codes [10, 11]. Such is the case with PFO; it shares the same ICD diagnostic code with atrial septal defects (ASD) (ICD-9 code 745.5 and ICD-10 code Q21.1), and in Ontario the same Canadian Classification of Interventions (CCI) code for closure via percutaneous intervention (1HN80GPFL), despite differences in clinical characteristics, indications to close both defects, and the device technologies to carry out these procedures [10, 11]. While PFO is considered a variant of normal structure, ASD results from abnormal development or damaging of the septum premium during embryological development [10]. Individuals with ASD tend to present with more comorbidities, which leads to comparatively less functionality; it is not uncommon for individuals to discover they have a PFO only after they have experienced a cryptogenic stroke [11, 15]. Furthermore, the lack of differentiation between these two patient groups becomes further problematic when taking into consideration the vast difference in prevalence; ASD is estimated to be present in approximately 0.05% of the population, compared to up to 25% for PFO [11].

In past attempts to separate PFO from ASD patient populations, the identification of PFO patients hinged primarily on a history of ischemic stroke within one year prior to the PFO closure (see Additional file 1: Appendix A for the summary of past attempts). A study by Merkler et al*.* utilized a more comprehensive classification method of isolating patients with PFO from other congenital heart diseases [8]. Patients were only included if there was a record of a TIA or ischemic stroke within one year prior to and during admission for PFO closure [8]. In addition, records with a rehabilitation diagnostic code (ICD-9: V57), trauma (ICD-9: 800–804 or 850–854), or intracerebral (ICD-9: 431) or subarachnoid (ICD-9: 430) hemorrhages were excluded [8]. Overall, two of the four identified studies used validated algorithms to identify stroke, TIA or congenital heart disease, but none were used to differentiate PFO and ASD specifically (Additional file 1: Appendix A). This study aimed to develop and validate a random forest classification algorithm to separate PFO and ASD patient populations with records of transcatheter closure within Ontario administrative data and enhance the use of administrative databases in future long-term outcome studies for these populations.

## Methods

### Health administrative data sources

This study utilizes a repository of Ontario’s administrative health databases housed at the ICES [14]. ICES is an independent, non-profit research institute whose legal status under Ontario’s health information privacy law allows it to collect and analyze health care and demographic data, without consent, for health system evaluation and improvement. These datasets were linked using unique encoded identifiers and analyzed at ICES. Since all Ontario residents are covered through a single-payer insurance system for physician, hospital-based care and home care services, and drugs for residents 65 years of age and older, healthcare encounters can be linked across systems through individual health card numbers and each resident receiving a unique ICES Key Number (IKN) [16]. Population-based ICES data sources linked for this analysis include the Canadian Institute for Health Information’s Discharge Abstract Database and Same Day Surgery database (CIHI-DAD/SDS), which report all hospital visits dated back to 1988, the CIHI National Ambulatory Care Reporting System (NACRS), which reports hospital and community-based ambulatory care visits starting from the year 2000, and the Ontario Health Insurance Plan (OHIP) database reporting outpatient physician services since 1991. The use of data in this project was authorized under Sect. 45 of Ontario’s Personal Health Information Protection Act, which does not require review by a Research Ethics Board.

### Cohort creation

Our study cohort comprised of all Ontario residents 18 years of age and older who had a transcatheter closure procedure for ASD or PFO closure recorded in CIHI-DAD/SDS (CCI code 1HN80GPFL) between October 2002 and December 2017.

### Reference standard database

CorHealth Ontario’s cardiac registry was selected as the reference standard [17, 18]. The CorHealth cardiac registry captures select clinical data on all cardiac procedures performed in Ontario catheterization laboratories [17, 18]. Two distinct fields in the catheterization laboratory data indicate if the procedure was a PFO closure (Yes/No) or an ASD closure (Yes/No), and were used in our study to identify if the procedure was closure of PFO, ASD, both or neither [19]. The index event date for each patient in the study sample was the date of the procedure.

If patients had multiple interventions, only the first intervention was kept for this analysis. Patient records were excluded from the study dataset if their ICES Key Number (IKN) was missing, invalid, or repeating, if their gender code was missing or invalid, if the patient was not a resident of Ontario, or if at the time of intervention, the patient was younger than 18 years of age. Records Cases labeled as having both PFO and ASD or neither diagnoses were excluded from the building of this classification algorithm.

### Algorithm variable selection and definitions

Variables extracted from ICES data were considered for inclusion into our algorithm to identify PFO cases based on clinical relevance and review of the literature. Please see Additional file 1: Appendix B for the full list of variables and their respective codes. Patient demographic information was captured through sex and age group. All of the following variables were reported during a 5-year lookback period prior to TC. History of stroke and TIA were available as dichotomous variables (i.e. presence/absence or yes/no flags) and total number of stroke or TIA events. An overall Charlson Comorbidity Index score was also retrieved from ICES [20]. Other comorbidities were defined ICD-based yes/no flags only. Healthcare utilization was captured by intervention codes reported during index admission, and any history of admission for ASD, PFO, or other congenital heart diseases (CHD) 5 years prior to closure.

### Random forest classification

Random forest models are made up of several decision trees, a non-parametric and supervised machine learning approach that may be used for both regression and classification tasks [21–23]. Decision trees are constructed by recursively splitting data based on simple rules learned from the input variables provided from a given dataset of interest [21–23]. With random forest models, each individual decision tree therein analyzes a different sample of the data, and then all trees “vote” as an ensemble what a given observation should be categorized as, in this case whether a patient has undergone transcatheter closure for a PFO or an ASD [21–23].

A random forest method was chosen because it is non-parametric and builds upon the positive attributes of the popular decision tree method such as providing implicit feature selection, and decreased sensitivity to outliers compared to other classification techniques such as logistic or linear regression [21, 23, 24]. Given the novel nature of this classification model, minimal a priori feature selection was preferred. Furthermore, by combining the results of multiple individual decision trees, it follows that a combination of all resultant outputs may result in a higher predictive accuracy than each constituent tree alone, especially with complex and high-dimensional data [23, 24]. The combination of this majority voting approach on sub-samples of the data is known as bootstrap aggregating, or bagging [21, 24]. Bagging decreases the likelihood of overfitting and improves model generalization by decreasing outlier influence and model variance [21, 24]. This then provides a unique advantage when encountering high-dimensional data with complex interactions [23, 24].

All versions of the classification model were run in R using the *randomForest* package with 500 trees generated within each random forest [25]. To assess model performance, the reference standard was randomly sampled and split 40/60 into a training and a test set. Performance measures were compared between test and training sets to assess models for degree of overfitting, i.e., if training values were much higher than test values. Overall model performance was based on test accuracy, sensitivity, and specificity.

Variable importance was assessed through a mean decrease in Gini index. The Gini index indicates a level of partition “purity” which the random forest model uses to determine its classifications [21, 23, 24]. The higher the mean decrease in Gini for a given variable, the less likely it is that variable will lead to misclassified patients across all constructed trees [21, 23, 24]. Variable importance scores were compared among covariates to determine their relative ranking.

The final model was chosen once performance measures were optimized via hyperparameter tuning of *mtry* and the decision threshold. *Mtry* is a hyperparameter that pertains to the randomness of the forest, namely how many of the variables are considered at each split [26]. To determine the correct value, a grid search was run with the *caret* package, where a linear search was performed for a vector of candidate *mtry* values, and the value resulting in the highest accuracy was used for the final model [27]. The classification threshold, at default set at 0.5, reflects the probability required for an observation, in this case a patient in the CorHealth dataset, to be classified as ASD or PFO [28]. Different values for this threshold were attempted until the resultant tuned model performance was optimized.

As a sensitivity analysis, model performance was also compared to prior classification methods, using the same reference data and performance measures, by designating patients who had experienced an ischemic stroke, a hemorrhagic stroke, or a TIA within 1 year of closure as PFO patients, and the rest as ASD patients, without using any machine learning methods. Please refer to Additional file 1: Appendix C for a reproducible example of utilized code with simulated data.

### Descriptive statistics of classified cohort

Following classification of patients by the random forest model as having undergone ASD or PFO transcatheter closure, the clinical and demographic characteristics were descriptively summarized in R by counts and percentages using the *tableone* package [29]. Clinical and demographic characteristics were compared between groups through chi-squared tests, with a significance level of *p* = 0.05.

## Results

### Study cohort creation

**Fig. 1:**
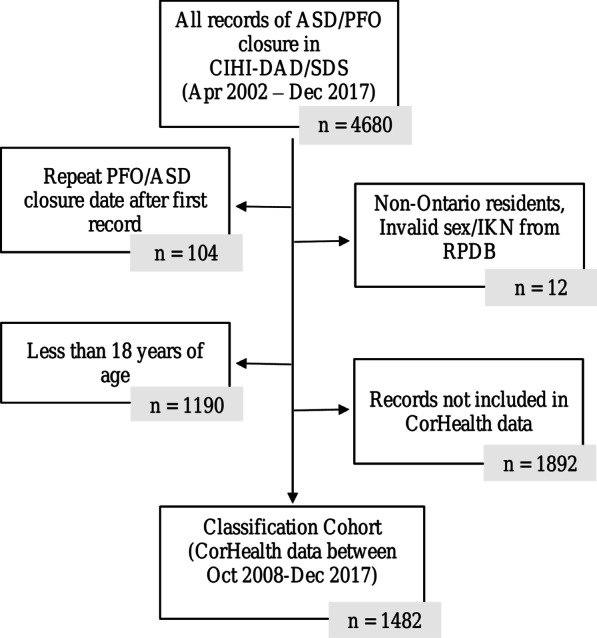
Exclusions from original ICES data linkage of transcatheter closures from CIHI-DAD/SDS to create study cohort (reference standard)

There were 4680 transcatheter closures performed in Ontario between April 1st, 2002 and December 31st, 2017 based on the CIHI-DAD/SDS database. After excluding any records of repeat closures (n = 104), non-Ontario residents, or those with invalid sex or IKN (n = 12), those less than 18 years of age (n = 1190), and any other records not having an ASD or PFO diagnosis from the CorHealth cardiac registry (n = 1892), our reference data comprised of 1482 patients (Fig. 1).

### Variables for classification

Clinical and demographic characteristics of the study cohort are presented in Table 1 and in more detail in Additional file 1: Appendix D. PFO and ASD patients were represented almost equally, with a slightly higher proportion of ASD population (PFO = 697, ASD = 785 patients). ASD patients were older and had more females than PFO patients. Among other differences in comorbidities, the proportion of patients with prior ischemic stroke events within 5 years of TC was higher in those with a PFO (39.5%) than in ASD (3.70%) patients.

**Table 1:** Demographic, Clinical Characteristics and secondary interventions of study cohort

	PFO (n = 697)	ASD (n = 785)	*P*-value
**Demographics**			
Sex (Female) ‒ n (%)	305 (43.8)	498 (63.4)	< 0.001
Age group ‒ n (%)			< 0.001
18–60	542 (77.8)	540 (68.8)	
> 60	155 (22.2)	245 (31.2)	
**Clinical characteristics**			
Ischemic stroke (total number ≥ 1)—n (%)	275 (39.5)	29 (3.7)	< 0.001
Hemorrhagic stroke (total number ≥ 1)—n (%)	< 6^1^	< 6^1^	0.600
TIA (total number ≥ 1)—n (%)	67 (9.6)	15 (1.9)	< 0.001
Other CHD hospitalizations—n (%)	144 (20.7)	167 (21.3)	0.821
Peripheral embolism, pulmonary embolism, or DVT—n (%)	40 (5.7)	13 (1.7)	< 0.001
Dyslipidemia—n (%)	< 6^1^	< 6^1^	1.000
Thrombophilia—n (%)	< 6^1^	< 6^1^	0.918
Migraine—n (%)	81 (11.6)	31 (3.9)	< 0.001
Renal failure—n (%)	12 (1.7)	32 (4.1)	0.012
AF—n (%)	50 (7.2)	120 (15.3)	< 0.001
CAD—n (%)	114 (16.4)	166 (21.1)	0.022
CHF—n (%)	34 (4.9)	63 (8.0)	0.019
COPD—n (%)	93 (13.3)	97 (12.4)	0.625
Diabetes—n (%)	72 (10.3)	106 (13.5)	0.073
HTN—n (%)	258 (37.0)	302 (38.5)	0.601
**Intervention codes**^**2**^			
Fluoroscopy, heart NEC without contrast—n (%)	20 (2.9)	26 (3.3)	0.734
**Xray**			
Thoracic cavity NEC—n (%)	41 (5.9)	17 (2.2)	< 0.001
Intravenous contrast injection, coronary veins—n (%)	127 (18.2)	118 (15.0)	0.114
Intraarterial contrast injection, pulmonary artery—n (%)	298 (42.8)	343 (43.7)	0.755
Intracardiac contrast injection, pulmonary artery	39 (5.6)	10 (1.3)	< 0.001
Steady state respiratory function study—n (%)	134 (19.2)	85 (10.8)	< 0.001
Heart capacity measurement, oxygen consumption technique—n (%)	123 (17.6)	129 (16.4)	0.581
Pressure measurement—n (%)	169 (24.2)	318 (40.5)	< 0.001
Ultrasound heart NEC, cardiac catheter inspection—n (%)	52 (7.5)	70 (8.9)	0.356
Heart and coronary artery ultrasound—n (%)	55 (7.9)	115 (14.6)	< 0.001

Ontario residents 18 years of age and older who had a transcatheter closure procedure for PFO or ASD between October 2002 and December 2017 (N = 1482) in the CorHealth Registry and CIHI Discharge Abstract Database and Same Day Surgery Database

*AF* atrial fibrillation, *CAD* coronary artery disease, *CHD* congenital heart disease, *CHF* congestive heart failure, *COPD* chronic obstructive pulmonary disease, *DVT* deep vein thrombosis, *HTN* hypertension, *NEC* not elsewhere classified by CCI/CCP codes, *TIA* transient ischemic attack

^1^Small cells (≤ 6 patients) were suppressed to comply with ICES privacy policies

^2^The 10 most frequent intervention codes beyond transcatheter closure

Before a final model was chosen and hyperparameters were tuned, many variable permutations were tested among 7 models in total, and modified based on model performance, with moderate performance overall ranging between 0.72 and 0.76 for accuracy, 0.51–0.64 for sensitivity, and 0.75–0.89 for specificity. Variables with highly skewed data, i.e. rare among this population, were excluded in some model versions. The final model, model 7 with tuned hyperparameters, contained 24 variables, of which all were dichotomous (yes/no) categorical variables except for age group (with total 13 age groups), Charlson comorbidity index (a numeric score), and total number of ischemic stroke and TIAs (Table 2). After hyperparameter tuning, model 7 achieved a test accuracy of 0.76, test sensitivity of 0.76, and test specificity of 0.75. Please see Additional file 1: Appendix E for detailed descriptions of all models, including both the final model and all remaining tested models.

**Table 2:** Description and performance of final random forest model to identify PFO patients

Model	Description		Accuracy		Sensitivity		Specificity	
			Test	Train	Test	Train	Test	Train
7	**Demographics** Age group Sex **Comorbidity flags (< 5 years)** AF CAD CHF COPD DM HTN Migraine Other CHD admissions Emb.*	**Stroke/TIA** Number of events < 5 years prior to closure Ischemic stroke Hemorrhagic stroke TIA **Intervention codes** Top 10 (yes/no) **Charlson comorbidity index**	0.946	0.756	0.908	0.657	0.978	0.848
7 (tuned)	Same variables as model 7 (above), but with hyperparameters tuned: mtry = 3 Classification threshold cut-off = 0.38,0.62		0.918	0.757	0.896	0.751	0.936	0.763

*Emb. peripheral arterial embolism, pulmonary embolism, or deep vein thrombosis

### Variable importance

**Fig. 2:**
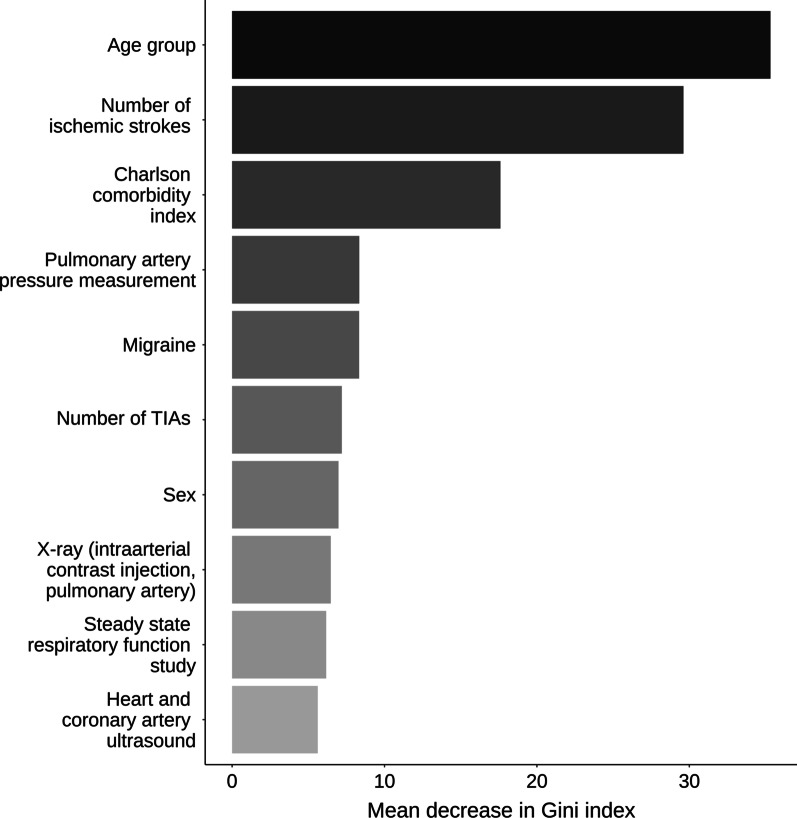
Variable importance graph, based on mean decrease in Gini index

For the final model, both age group (~ 35% mean decrease in Gini) and the count of ischemic strokes in the 5 years prior to closure (~ 30% mean decrease in Gini) contributed the most to the partitioning of the data (Fig. 2). Of all individual intervention codes, the code indicating a test for pulmonary artery pressure measurement had the highest mean decrease in Gini index. Charlson comorbidity index, history of migraines, and sex contributed the most to the accuracy of this model compared to the other variables.

#### Model performance and comparison to traditional method

After selecting the best-performing version of the model, model 7, sensitivity was improved further through hyperparameter tuning of *mtry* and the classification threshold, with some cost to the specificity. The results of the final random forest model, its improved tuned version, and the performance of the traditional classification method are summarized in Table 3. For all versions of the random forest model, the majority had a moderate accuracy, low sensitivity, and high specificity (Table 3, Additional file 1: Appendix E). The traditional model, not using machine learning methods, also had a moderate but lower accuracy score (0.68), and a high specificity (0.96), but a very low sensitivity (0.36) compared to both the tuned and untuned final random forest model (Table 3).

**Table 3:** Comparison of classification performance between the “traditional model”, versus the random forest model that considered additional variables

Model	Test accuracy	Test sensitivity	Test specificity
Traditional*	0.68	0.36	0.96
Final random forest model (original)	0.76	0.66	0.85
Final random forest model (tuned)	0.76	0.75	0.76

*Patients were assigned as a PFO based only on ‘any stroke or TIA within 1 year of closure’, and if not, they were assigned as ASD

## Discussion

In this study, a random forest model was built by combining a wide array of comorbidity and demographic information from health administrative data to classify patients as undergoing transcatheter closure of PFO or ASD. To our knowledge, it is the first study to utilize machine learning techniques to separate these patient groups in administrative data. Using the CorHealth cardiac registry as the reference standard, our model achieved an overall accuracy of 76% with balanced sensitivity (76%) and specificity (75%), which is a much better and more balanced classification performance than the traditional approach which identifies a TC for PFO or ASD based only on past history of stroke or TIA (accuracy 68%, sensitivity 36%, and specificity 96%).

Our random forest model identified several additional variables that can be used to improve the overall performance of PFO and ASD classification. Although the final model ended up with a relatively large number of variables, comorbidities and intervention codes should be easily available in similar administrative databases. The specific intervention codes that were most common within this patient population may differ between different administrative data systems, but should still be easily available as billable codes.

A different type of administrative data was used as the reference standard in our study due to its wide availability. CorHealth registry data is collected by hospitals, where all TC procedures are performed and includes funding for clinical abstractors, primarily for quality assurance and accountability rather than for billing purposes. Although ICES administrative databases had data available starting in 2002, CorHealth data was available only from 2008 to 2017. While the lack of overlap between 2002 and 2008 may have resulted in some loss of information, we believe that the available data provided sufficient information to train the classification model. Furthermore, although reliability and validity checks are not routine for CorHealth data aside from identifying any missing data, the clinical richness and attention to distinguishing between TC for PFO and ASD makes it an acceptable reference standard to assess the accuracy of an administrative database algorithm.

With regards to the use of other administrative databases, while the coding within CIHI-DAD has been found to be very accurate for procedural information and individual demographics, its quality is considerably more variable for coding of major diagnoses [30, 31]. They are also not reported for the purpose of research, and so may not describe clinical information to the degree of detail as may be desired by some research studies. In addition, ICES databases do not capture health care usage outside of Ontario [30, 31]. Although PFO and ASD do not have their own distinct ICD codes, most variables in this dataset used to build the random forest model, as with all administrative health data, hinge on the usage of ICD codes. Generally, diagnostic codes can carry a risk of inaccurate indication of true disease status, leading to potential misclassification bias [32]. Regardless of the personal knowledge of the individual entering the information, the inherent structure of ICD codes may not allow the inclusion of important clinical details such as any underlying anatomic diagnoses or a history of interventions or surgeries that may be pertinent to long-term patient health outcomes [11]. Furthermore, there is little to no quality control to confirm accurate coding on the level of individual patients [11]. This can provide challenges in describing and differentiating pathologies of interest when based on ICD codes alone [10, 11].

Given these known challenges with administrative data, certain performance measures were chosen to make this model useful for a wide variety of purposes. The eventual intention of creating this classification model was to utilize it for studying long-term outcomes for both ASD and PFO patients, allowing for comparison between different health care sites, which has not been feasible thus far using administrative data. As such, classification algorithms should target identifying both patient groups rather than prioritizing identification of one over the other. This is why sensitivity, specificity, and accuracy were evaluated together rather than focusing on one specific measure. Typically, sensitivity is prioritized when the primary consideration is to identify true positives, even at the risk of including false positives [33]. This is done to enhance the inclusiveness of the model, and can improve the generalizability of the results [33, 34]. To balance this, and because it is inversely related to sensitivity, specificity is evaluated as well to identify true negatives [35]. Although positive predictive value is often prioritized when identifying a patient cohort, accuracy was chosen in this study as the overall measure of how well the model differentiated between transcatheter procedure done for PFO and ASD patients [36]. Because the correct identification of both PFO and ASD patients were of chief interest for this study, it was important to take into account not only overall performance of the model through accuracy, but also keep in mind the balance between sensitivity and specificity.

There are some limitations related specifically to the use of random forest for classification. Random forest models, like other means of predictive algorithmic modeling, are probability-based and data-driven. As such, although the modeling strategy may be transferred over to different settings, the specific model may not be directly applied, and so external validation is necessary prior to future usage [37, 38]. A second set of labeled data was unavailable for our study, so external validation was not possible in this case. Investigation of potential variable interactions could also provide an area for future work. Additionally, while the complexity of random forests makes them a powerful data modeling tool, they are less easily interpretable than other models and so may not be as accessible in certain settings [37, 38]. This includes the lack of e.g. regression coefficients, however variable importance data output by the random forest model aids in interpretability and may operate in much the same way.

## Conclusions

This study has demonstrated moderate accuracy of an administrative database algorithm to identify PFO or ASD diagnosis. Our random forest classification model found a history of stroke/TIA, as well as other comorbidities, improved the accuracy of determining whether transcatheter closure was performed on a PFO or ASD. External validation of our algorithm in other administrative databases or another reference standard is recommended to determine the generalizability of our algorithm.

## Supplementary Information

**Additional file 1**.** Appendix A** - Prior attempts in literature to differentiate PFO from ASD and other congenital heart diseases; **Appendix B** - Diagnostic and procedural codes used to define baseline comorbidities; **Appendix C** - Reproducible example with simulated data; **Appendix D** - Detailed table of baseline demographic information and comorbidities; **Appendix E** - Models tested to determine final classification algorithm.

## Data Availability

The dataset contains individual-level sensitive health information, and so is held securely in coded form at ICES. While data sharing agreements prohibit ICES from making the dataset publicly available, access may be granted to those who meet pre-specified criteria for confidential access, available at www.ices.on.ca/DAS. Although due to these regulations the data itself cannot be shared by the authors, the full dataset creation plan and underlying analytic code are available from the authors upon request, understanding that the computer programs may rely upon coding templates or macros that are unique to ICES and are therefore either inaccessible or may require modification.

## Abbreviations

AF: Atrial fibrillation
ASD: Atrial septal defects
CAD: Coronary artery disease
CCI: Canadian Classification of Interventions
CHD: Congenital heart disease
CHF: Congestive heart failure
CIHI: Canadian Institute for Health Information
COPD: Chronic obstructive pulmonary disease
CS: Cryptogenic stroke
DAD: Discharge Abstract Database
DVT: Deep vein thrombosis
ED: Emergency department
HR: Hazard ratio
HTN: Hypertension
ICD: International Statistical Classification of Diseases
IKN: ICES Key Number
MOHLTC: Minister of Health and Long-Term Care
NACRS: National Ambulatory Care Reporting System
OHIP: Ontario Health Insurance Plan
PFO: Patent foramen ovale
RCTs: Randomized controlled trials
SDS: Same Day Surgery
TC: Transcatheter closure
TIA: Transient ischemic attack
