# Supplementary material for: Developing a random forest algorithm to identify patent foramen ovale and atrial septal defects in Ontario administrative databases
Source: BMC Med Inform Decis Mak. 2022 Apr 6;22:93. doi: 10.1186/s12911-022-01837-2 (PMC8988372; doi:10.1186/s12911-022-01837-2)
Supplement: Supplementary file 1 — Additional file 1. Appendix A - Prior attempts in literature to differentiate PFO from ASD and other congenital heart diseases; Appendix B - Diagnostic and procedural codes used to define baseline comorbidities; Appendix C - Reproducible example with simulated data; Appendix D - Detailed table of baseline demographic information and comorbidities; Appendix E - Models tested to determine final classification algorithm. [file 12911_2022_1837_MOESM1_ESM.pdf]

APPENDICES

Appendix A – Prior attempts in literature to differentiate PFO from ASD and other congenital heart diseases

**Study**<br>***First Author (Year)***	**Country**	**Data source**	**PFO identification**	**Other CHD identification**
**Kotowycz (2013)** [156]	Canada	Québec CHD Database	• PFO/ASD diagnosis<br>- ICD-9: 745.5<br>• PFO closure based on any of the following ≤ 1 year prior to closure:<br>- Stroke (ICD-9: 435.9)<br>- TIA (ICD-9: 435.9)	**Group:** Secundum ASD patients that underwent closure (TC/surgical)<br>• PFO/ASD diagnosis<br>ICD-9: 745.5
**Mylotte (2013)** [157]	Canada	Québec CHD Database	• PFO/ASD diagnosis<br>- ICD-9: 745.5<br>• Stroke ≤ 1 year prior to closure<br>*ICD code(s) NR*	**Group:** Secundum ASD patients that underwent closure (TC/surgical)<br>• PFO/ASD diagnosis<br>ICD-9: 745.5
**Lanz (2015)** [158]	Canada	Québec CHD Database	Record of the following ≤ 1 year prior to first-ever record of closure (ICD-9: 745.5, ICD-10: Q21.1)<br>• Ischemic stroke<br>- ICD-9: 434,436<br>- ICD-10: I63, I64<br>• Hemorrhagic stroke<br>- ICD-9: 431<br>- ICD-10: I61<br>• TIA<br>*ICD code(s) not reported*	**Group:** Secundum ASD patients that underwent closure (TC/surgical)<br>• PFO/ASD diagnosis<br>ICD-9: 745.5
**Merkler (2017)** [49]	USA	Administrative claims data on all hospitalizations from 2005–2013 in New York, California, and Florida	First recorded hospitalization for TC<br>- ICD-9: 35.52<br>Either of the following ≤ 1 year before/during this first hospitalization<br>• Ischemic stroke (ICD-9: 433.1,434.1,436) unaccompanied by:<br>- Primary discharge code for rehabilitation (ICD-9: V57)<br>- Trauma (ICD-9: 800–804 or 850–854)<br>- Intracerebral hemorrhage (ICD-9: 431)<br>- Subarachnoid hemorrhage (ICD-9: 430)<br>• TIA (ICD-9: 435)	**Group:** Non-PFO TC for CHD<br>• Previous or concurrent documented history of CHD<br>ICD-9: 745.1-745.4, 745.6-745.8,746,747

ASD = atrial septal defect, NR = not reported, PFO = patent foramen ovale, CHD = congenital heart disease, TC = transcatheter closure, TIA = transient ischemic attack, RLS = right-to-left shunt.

Appendix B – Diagnostic and procedural codes used to define baseline comorbidities

Variable	CIHI DAD/NACRS/SDS (ED or Inpatient diagnostic or procedure* codes)	OHIP (Physician claim diagnostic codes)	References/Notes
Arterial embolism and thrombosis	**ICD9:** 444.21, 444.22<br>**ICD10:** I74		
Atrial fibrillation (AF)	**ICD9: 427**.3<br>**ICD10:** I480, I4890<br>Rule: 1 DAD code in the past two years	427, Z437	[159]
Atrial septal aneurysm	**ICD9: 414**.1<br>**ICD10:** I25.3		
Atrial septal defect (ASD)/ patent foramen ovale (PFO)	**ICD9:** 745.5<br>**ICD10:** Q21.1		
Coronary artery disease (CAD)	**ICD9:** 410-414<br>**ICD10:** I20, I21, I22, I23, I24, I25<br>**CCI:** 1IJ50, 1IJ57, 1IJ76<br>**CCP:**481, 4802, 4803, 4809	410, 412, 413, Z434, G298, R742, R743	[160]
Cardioversion		Z437	
Chronic obstructive pulmonary disorder (COPD)	**ICD9:** 491, 492, 496<br>**ICD10:** J41, J42, J43, J44	491, 492, 496	ICES-derived cohort<br><br>Rule: three or more OHIP codes and/or one or more DAD code within two years (1991 to present).
Congestive heart failure (CHF)	**ICD9:** 428<br>**ICD10:** I50	428	[161]<br><br>Rule: 1 NACRS, DAD, SDS, or OHIP claim and a second claim (from either) in 1 year (1991 to present) or any 1 DAD record
Deep vein thrombosis (DVT)	**ICD9:** 451.1x, 451.2, 451.81, 453.4x, 453.5x<br>**ICD10:** I80.1–I80.9, I82.1, I82.8, I82.9, O22.3, O22.9, and O87.1		[162, 163]
Diabetes	**ICD-9**: 250<br>**ICD-10**: E10, E11, E13, E14	250, Q040, K029, K030, K045, K046	ICES-derived cohort<br>Rule: two OHIP diagnostic codes or 1 OHIP service code or 1 DAD/SDS code within 2 years (1991 to present)
Dyslipidemia	**ICD9:** 272<br>**ICD10:** E78		
Holter monitoring<br>Short-term<br>Medium-term<br>Long-term		 G650, G658<br>G659<br>G649	
Hypertension	**ICD9:** 401, 402, 403 404, 405<br>**ICD10:** I10, I11, I12, I13, I15	401, 402, 403 404, 405	ICES-derived cohort<br>Rule: 1 DAD/SDS or 1 OHIP claim followed within two years by either an OHIP claim or a DAD claim (1991 to present).
Malignancy	**ICD9:** 140-208<br>**ICD10:** C00-C97		Rule: 1 DAD code in the past 2 years.
Migraine	**ICD9:** 346, 784, 349, 307.8, 627.2, 296.2, 296.3, 298.0, 300.4<br>**ICD10:** G43, G44, R51, G91.1, N95.1, F32, F33, F34.1	346	[164, 165]

Variable	CIHI DAD/NACRS/SDS (ED or Inpatient diagnostic or procedure* codes)	OHIP (Physician claim diagnostic codes)	References/Notes
Mortality			
All-cause	**ICD9:** 390-434, 436-448, 001-389, 460-676, 680-999, E800-E999, V01-V82<br>**ICD10:** I00-I79, A00-D48, D50-D89, E00-E90, F00-H95, J00-K93, L00-P96, Q00-T98, V01-Y98, Z00-Z99, U00-U99		
Cardiovascular-related	**ICD9:** 390-434, 436-448<br>**ICD10:** I00-I79		
Myocardial infarction (MI)	**ICD9:** 410I<br>**ICD10:** I21, I22		Rule: 1 DAD code in the past two years.
Other congenital heart disease	**ICD9:** 745-747 (except 745.5)<br>**ICD10:** Q20-Q28 (except Q21.1)		
Pacemaker implantation	**CCI:**1HB53, 1HZ53, 1HD54, 1HD53<br>**CCP:** 49.7, 49.71, 49.72, 49.73, 49.74, 49.84, 49.83		
Pulmonary embolism	**ICD9:** 415.1x<br>**ICD10:** I26.0–I26.9, O88.2		[162, 163]
Renal failure	**ICD9:** 585, 586<br>**ICD10:** E102, E112, E132, E142, I12, I13, N08, N18, N19		Rule: 1 DAD code in the past 2 years.
Stroke, ischemic	**ICD9:** 434, 436, 362.3<br>**ICD10:** I63 (excluding I63.6), I64, H34.1		Rule: 1 DAD code in the past 2 years.
Stroke, hemorrhagic	**ICD9:** 430, 431<br>**ICD10:** I60, I61		Rule: 1 DAD code in the past 2 years.
Thrombophilia	**ICD9:** 289.81, 289.82<br>**ICD10:** D68.5, D68.6		[166]
Transcatheter PFO/ASD closure	**CCI**: 1HN80GPFL/1HN80GPG<br>**CCP:** 47.53		
Transient ischemic attack (TIA)	**ICD9:** 435<br>**ICD10:** G450, G451, G452, G453, G458, G459, H34.0		

CCI = Canadian Classification of Health Interventions, CCP = Canadian Classification of Diagnostic, Therapeutic and Surgical Procedures, CIHI = Canadian Institute of Health Information, DAD = Discharge Abstract Database,ICD= International Statistical Classification of Diseases, *NACRS = National Ambulatory Care Reporting System, OHIP = Ontario Health Insurance Plan, SDS = Same Day Surgery*

Appendix C - Reproducible example with simulated data

I. Setup - upload your data and necessary libraries

***Sim_data* is a dataset of 28 variables with the same names and R data type (i.e. factor, integer, or numeric) as the comorbidity and demographic variables used in this study. In practice, the variables can be of any R data type appropriate to your dataset of interest. 2000 observations were randomly generated for each variable. "`PFO_ASD`" is our classifier variable.**

Make sure the simulated data (sim_data.Rdata) is in your working directory prior to loading.

```
load("sim_data.Rdata")
library(randomForest)
library(epiR)
library(caret)
library(dplyr)
```

When creating your classification dataset, include only the variables you intend on using as covariates in your classification model. Check that they are in the correct data format (e.g. a yes/no flag indicated by 1/0 should be coded as a "factor" with two levels, 1 and 0, rather than as numeric) and that there is no missing data – the *randomForest* package does not work in the presence of missing data, even if the NAs are within variables not specified by the model equation when you run *randomForest*. Incorrect data formats can also affect the performance of your model if they are unknowingly imported into R as the incorrect format.

II. Create training and test datasets

For this study, the data was split into a 40/60 train/test split.

```
set.seed(123)
train.sample <- sample(2,
                       nrow(sim.data),
                       replace = T,
                       prob = c(0.40,0.60))

train.df = sim.data[train.sample==1,]
test.df = sim.data[train.sample==2,]
```

III. Hyperparameter tuning - determine optimal value of mtry (i.e. the number of variables tried at each split

i. Run grid search

```
control <- trainControl(method="repeatedcv",
                        number=10,
                        repeats=3)
set.seed(123)
tunegrid <-expand.grid(.mtry=c(1:15))
rf_gridsearch <- train(PFO_ASD~.,data=train.df,method="rf",metric="Accuracy",
tuneGrid=tunegrid, trControl=control)
```

To run our grid search, we need to input our model equation. PFO_ASD is our classifier variable. "`~.`" tells it to include all other variables as covariates in the classification.

ii. Output results of grid search

```
print(rf_gridsearch)
```

Whichever value for *mtry* provides the highest accuracy is what you will input in the model later.

```
Random Forest

779 samples
 34 predictor
  2 classes: 'ASD', 'PFO'

No pre-processing
Resampling: Cross-Validated (10 fold, repeated 3 times)
Summary of sample sizes: 701, 701, 701, 701, 701, 702, ...
Resampling results across tuning parameters:

  mtry  Accuracy   Kappa
   1    0.5002772  -0.008987576
   2    0.5144350   0.025584191
   3    0.5061895   0.010269761
   4    0.5122380   0.023043750
   5    0.5143852   0.027213521
   6    0.5143583   0.027685708
   7    0.5217064   0.042664309
   8    0.5216459   0.042468953
   9    0.5241281   0.047307611
  10    0.5229665   0.045279970
  11    0.5152628   0.029867020
  12    0.5229286   0.045267699
  13    0.5259031   0.051110077
  14    0.5272787   0.054152573
  15    0.5272620   0.053879006

Accuracy was used to select the optimal model using the largest value.
The final value used for the model was mtry = 14.
```

```
plot(rf_gridsearch)
```

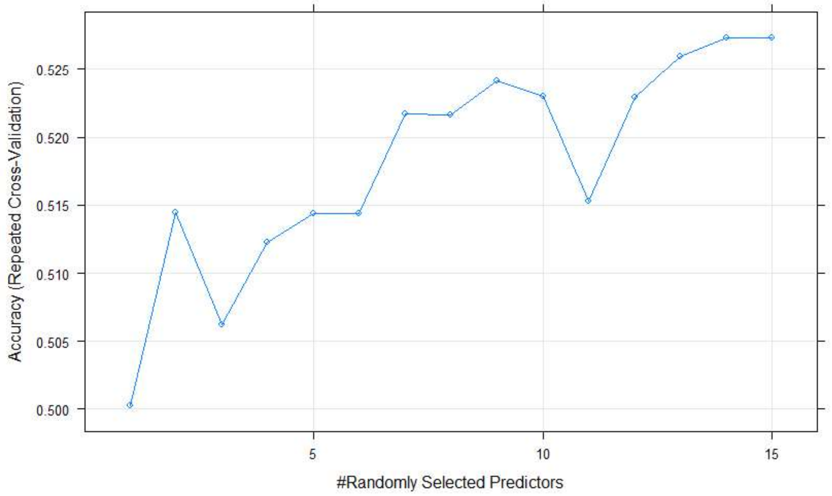

IV. Run random forest model

Although we have a way to determine the optimal value of mtry, the <u>classification threshold</u> is selected by trial and error (i.e. re-running the model with different threshold values until performance is sufficiently improved). The cutoff values must also add up to 1.

```
rf = randomForest(PFO_ASD ~.,
                  ntree=500,
                  cutoff=c(0.45,0.55),
                  mtry=14,
                  data=train.df)
```

Our model equation

Number of trees in your random forest

Classification threshold

Training dataset

Mtry value from grid search

```
print(rf)

##
## Call:
##  randomForest(formula = PFO_ASD ~ ., data = train.df, ntree = 500,      cu
toff = c(0.45, 0.55), mtry = 14)
##                Type of random forest: classification
##                      Number of trees: 500
## No. of variables tried at each split: 14
##
##         OOB estimate of  error rate: 53.79


## Confusion matrix:
##     ASD PFO class.error
```

```
## ASD 239 146   0.3792208
## PFO 273 121   0.6928934
```

The default model output gives you a look at the confusion matrix, but this is not very informative – which is why the performance measures are calculated next.

V. Determine predicted response for train and test data

This creates a column in your training and test data with the predicted responses. Make sure if you are re-running the model to remove these "`predicted.response`" columns first from the training and test datasets.

```
train.df$predicted.response=predict(rf,train.df)

test.df$predicted.response=predict(rf,test.df)
```

VI. Calculate performance measures

Because the simulated data is completely random, the model performance for the training set will be very high, and for the test set it will be poor.

Training set

```
print(
confusionMatrix(data = train.df$predicted.response,
                reference = train.df$PFO_ASD,
                positive = 'PFO'))

## Confusion Matrix and Statistics
##
##           Reference
## Prediction ASD PFO
##        ASD 385   0
##        PFO   0 394
##
##                Accuracy : 1
##                  95 CI : (0.9953, 1)
##     No Information Rate : 0.5058
##     P-Value [Acc > NIR] : < 2.2e-16
##
##                   Kappa : 1
##
##  Mcnemar's Test P-Value : NA
##



##
##             Sensitivity : 1.0000
##             Specificity : 1.0000
```

```
##          Pos Pred Value : 1.0000
##          Neg Pred Value : 1.0000
##              Prevalence : 0.5058
##          Detection Rate : 0.5058
##    Detection Prevalence : 0.5058
##       Balanced Accuracy : 1.0000
##
##        'Positive' Class : PFO
##
```

Test Set

```
print(
confusionMatrix(data = test.df$predicted.response,
                reference = test.df$PFO_ASD,
                positive = 'PFO'))
```

```
## Confusion Matrix and Statistics
##
##           Reference
## Prediction ASD PFO
##        ASD 388 350
##        PFO 246 237
##
##                Accuracy : 0.5119
##                  95 CI : (0.4834, 0.5403)
##     No Information Rate : 0.5192
##     P-Value [Acc > NIR] : 0.7069
##
##                   Kappa : 0.0158
##
##  Mcnemar's Test P-Value : 2.453e-05
##
##             Sensitivity : 0.4037
##             Specificity : 0.6120
##          Pos Pred Value : 0.4907
##          Neg Pred Value : 0.5257
##              Prevalence : 0.4808
##          Detection Rate : 0.1941
##    Detection Prevalence : 0.3956
##       Balanced Accuracy : 0.5079
##
##        'Positive' Class : PFO
##
```

VII. Variable importance

```r
varImpPlot(rf,
           sort=T,
           n.var = 10,
           main = "Top 10 - Variable Importance")
```

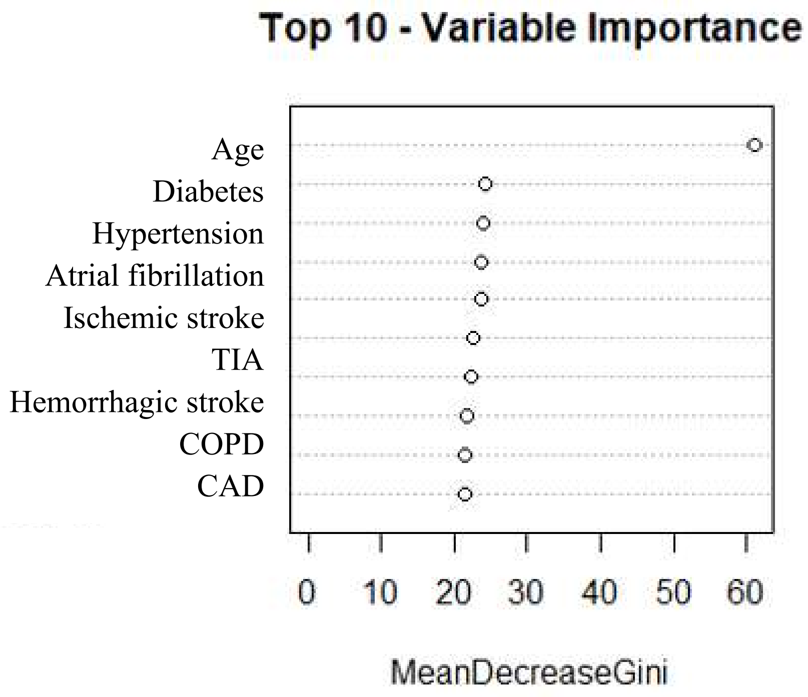

Appendix D – Detailed table of baseline demographic information and comorbidities

	PFO (N =697)	ASD (N =785)	P-VALUE
**DEMOGRAPHICS**			
Sex, (Female) – n (%)	305 (43.8)	498 (63.4)	<0.001
Age group – n (%)			<0.001
18-20	13 (1.9)	33 (4.2)	
21-25	19 (2.7)	49 (6.2)	
26-30	32 (4.6)	47 (6.0)	
31-35	40 (5.7)	63 (8.0)	
36-40	57 (8.2)	51 (6.5)	
41-45	99 (14.2)	88 (11.2)	
46-50	107 (15.4)	73 (9.3)	
51-55	95 (13.6)	73 (9.3)	
56-60	80 (11.5)	63 (8.0)	
61-65	48 (6.9)	77 (9.8)	
66-70	44 (6.3)	53 (6.8)	
71-75	29 (4.2)	50 (6.4)	
76+	34 (4.9)	65 (8.3)	
**CLINICAL CHARACTERISTICS**			
History of stroke/TIA <5 years prior to closure – n (%)			
Any stroke (ischemic, hemorrhagic, or TIA)	322.0 (46.2)	41.0 (5.2)	<0.001
Ischemic stroke	275.0 (39.5)	29.0 (3.7)	<0.001
Hemorrhagic stroke	< 6[1]	< 6[1]	0.600
TIA	68.0 (9.8)	15.0 (1.9)	<0.001
Number of stroke/TIA events – mean (SD)			
Ischemic stroke	0.7 (1.01)	0.06 (0.37)	<0.001
Hemorrhagic stroke	0.01 (0.165)	0.006 (0.11)	0.371
TIA	0.1 (0.406)	0.02 (0.17)	<0.001
Charlson Comorbidity Index – mean (SD)	1.0 (1.29)	0.5 (1.09)	<0.001
Other CHD hospitalizations – n (%)	144.0 (20.7)	167.0 (21.3)	0.821
Peripheral embolism, pulmonary embolism, or DVT – n (%)	40.0 (5.7)	13.0 (1.7)	<0.001
Dyslipidemia – n (%)	< 6[1]	< 6[1]	1.000
Thrombophilia – n (%)	< 6[1]	< 6[1]	0.918
Migraine – n (%)	81.0 (11.6)	31.0 (3.9)	<0.001
Renal Failure – n (%)	12.0 (1.7)	32.0 (4.1)	0.012
AF – n (%)	50. (7.2)	120.0 (15.3)	<0.001
CAD – n (%)	114.0 (16.4)	166 (21.1)	0.022
CHF – n (%)	34.0 (4.9)	63.0 (8.0)	0.019
COPD – n (%)	93.0 (13.3)	97.0 (12.4)	0.625
Diabetes – n (%)	72.0 (10.3)	106.0 (13.5)	0.073
HTN – n (%)	258.0 (37.0)	302.0 (38.5)	0.601
Intervention codes[2] – n (%)			
Fluoroscopy, heart NEC* without contrast, (Yes) – n (%)	20 (2.9)	26 (3.3)	0.734
Xray			
Thoracic cavity NEC, (Yes) – n (%)	41 (5.9)	17 (2.2)	<0.001
Intravenous contrast injection, coronary veins, (Yes) – n (%)	127 (18.2)	118 (15.0)	0.114
Intraarterial contrast injection, pulmonary artery, (Yes) – n (%)	298 (42.8)	343 (43.7)	0.755
Intracardiac contrast injection, pulmonary artery, (Yes) – n (%)	39 (5.6)	10 (1.3)	<0.001
Steady state respiratory function study, (Yes) – n (%)	134 (19.2)	85 (10.8)	<0.001
Heart capacity measurement, (Yes) – n (%)	123 (17.6)	129 (16.4)	0.581
Pressure measurement, (Yes) - n (%)	169 (24.2)	318 (40.5)	<0.001
Ultrasound heart NEC, cardiac catheter inspection	52 (7.5)	70 (8.9)	0.356
Heart and coronary artery ultrasound, (Yes) – n (%)	55 (7.9)	115 (14.6)	<0.001
Number of intervention codes – n (%)			<0.001
≤ 1	422 (60.5)	405 (51.6)	
> 1	275 (39.5)	380 (48.4)	
Total count of intervention codes* – mean (SD)	1.52 (1.2)	1.57 (1.06)	0.387

AF = atrial fibrillation, CAD = coronary artery disease, CHF = congestive heart failure, COPD = chronic obstructive pulmonary disease, DVT = deep vein thrombosis, NEC = not elsewhere classified, TIA = transient ischemic attack, SD = standard deviation

[1]Small cells (≤ 6 patients) were suppressed to comply with ICES privacy policies.

[2]The top 10 most frequently reported intervention codes aside from those for transcatheter closure

Appendix E – Models tested to determine final classification algorithm

Model	Description	Accuracy		Sensitivity		Specificity	
		Train	Test	Train	Test	Train	Test
1	*All comorbidity data*<br>**Demographics**<br>• Age group<br>• Sex<br>**Comorbidity flags (<5 years)**<br>• AF<br>• CAD<br>• CHF<br>• COPD<br>• DLP<br>• DM<br>• Emb*<br>• HTN<br>• Migraine<br>• Other CHD admissions<br>• RF<br>• Thrombophilia<br>**Stroke/TIA**<br>• < 5 years prior to closure (yes/no)<br>- Any stroke<br>- Ischemic stroke<br>- Hemorrhagic stroke<br>- TIA<br>**Intervention codes**<br>• Top 10 (yes/no)<br>**Charlson comorbidity index**	0.932	0.759	0.873	0.634	0.981	0.875
2	*Only comorbidities and intervention codes that were statistically significant between ASD/PFO groups*<br>**Demographics**<br>• Age group<br>• Sex<br>**Comorbidity flags (<5 years)**<br>• AF<br>• CAD<br>• CHF<br>• Emb.<br>• Migraine<br>• RF<br>**Stroke/TIA**<br>• < 5 years prior to closure (yes/no)<br>- Any stroke<br>- Ischemic stroke<br>- TIA<br>**Intervention codes**<br>• Top 10 (yes/no)<br>- Steady state respiratory function study<br>- Pressure measurement<br>- Intracardiac contrast injection, pulmonary artery<br>- Heart and coronary artery ultrasound<br>**Charlson comorbidity index**	0.843	0.727	0.692	0.563	0.968	0.879

Model	Description	Accuracy		Sensitivity		Specificity	
		Train	Test	Train	Test	Train	Test
**3**	*All comorbidity data but with changes to stroke/TIA and intervention codes*<br>**Demographics**<br>• Age group<br>• Sex<br>**Comorbidity flags (<5 years)**<br>• AF<br>• CAD<br>• CHF<br>• COPD<br>• DLP<br>• DM<br>• Emb<br>• HTN<br>• Migraine<br>• Other CHD admissions<br>• RF<br>• Thrombophilia<br>**Stroke/TIA**<br>• < 5 years prior to closure (yes/no)<br>- Any stroke<br>- Hemorrhagic stroke<br>- Ischemic stroke<br>- TIA<br>**Intervention codes**<br>• ≤ 1 vs > 1 reported intervention codes (yes/no)<br>**Charlson comorbidity index**	0.841	0.723	0.685	0.547	0.971	0.886
**4**	*Only comorbidities that were significant between PFO/ASD groups, except with intervention code counts instead of individual variables*<br>**Demographics**<br>• Age group<br>• Sex<br>**Comorbidity flags (<5 years)**<br>• Migraine<br>• RF<br>• CAD<br>• CHF<br>• AF<br>**Stroke/TIA**<br>• < 5 years prior to closure (yes/no)<br>- Any stroke<br>- Hemorrhagic stroke<br>- Ischemic stroke<br>- TIA<br>• Number of events < 5 years prior to closure<br>- Ischemic stroke<br>- Hemorrhagic stroke<br>- TIA<br>**Intervention codes**<br>• ≤ 1 vs > 1 reported intervention codes (yes/no)<br>**Charlson comorbidity index**	0.797	0.715	0.596	0.508	0.965	0.907
**5**	*All comorbidities except rare ones (thrombophilia, DLP, RF, Emb., hemorrhagic stroke), and keep ischemic stroke and TIA only, formatted as dichotomous variables*<br>**Demographics**<br>• Age group<br>• Sex<br>**Comorbidity flags (<5 years)**<br>• AF<br>• CAD<br>• CHF<br>• COPD<br>• DM<br>• HTN<br>• Migraine<br>• Other CHD admissions<br>**Stroke/TIA**<br>• < 5 years prior to closure (yes/no)<br>- Ischemic stroke<br>- TIA<br>**Intervention codes**<br>• Top 10 (yes/no)<br>**Charlson comorbidity index**	0.937	0.753	0.888	0.636	0.978	0.860

Model	Description	Accuracy		Sensitivity		Specificity	
		Train	Test	Train	Test	Train	Test
**6**	*All comorbidities except rare ones (thrombophilia, DLP, RF, Emb., hemorrhagic stroke), and keep ischemic stroke and TIA only, formatted as total counts*<br>**Demographics**<br>• Age group<br>• Sex<br>**Comorbidity flags (<5 years)**<br>• AF<br>• CAD<br>• CHF<br>• COPD<br>• DM<br>• HTN<br>• Migraine<br>• Other CHD admissions<br>**Stroke/TIA**<br>• Number of events < 5 years prior to closure<br>- Ischemic stroke<br>- TIA<br>**Intervention codes**<br>• Top 10 (yes/no)<br>**Charlson comorbidity index**	0.934	0.745	0.873	0.620	0.984	0.860
**7**	*Add rare comorbidities back in that are statistically significant between PFO/ASD groups (just Emb.), keep stroke/TIA as counts*<br>**Demographics**<br>• Age group<br>• Sex<br>**Comorbidity flags (<5 years)**<br>• AF<br>• CAD<br>• CHF<br>• COPD<br>• DM<br>• HTN<br>• Migraine<br>• Other CHD admissions<br>• Emb.<br>**Stroke/TIA**<br>• Number of events < 5 years prior to closure<br>- Ischemic stroke<br>- TIA<br>**Intervention codes**<br>• Top 10 (yes/no)<br>**Charlson comorbidity index**	0.939	0.757	0.885	0.638	0.984	0.867
**7 (tuned)**	*The same variables as Model 7 (above), but with hyperparameters tuned as follows:*<br>**Mtry** = 3<br>**Classification threshold** = 0.38,0.62	0.927	0.756	0.919	0.760	0.933	0.753
